# Geographic Expansion of Buruli Ulcer Disease, Cameroon

**DOI:** 10.3201/eid1703091859

**Published:** 2011-03

**Authors:** Estelle Marion, Jordi Landier, Pascal Boisier, Laurent Marsollier, Arnaud Fontanet, Philippe Le Gall, Jacques Aubry, Noumen Djeunga, Alphonse Umboock, Sara Eyangoh

**Affiliations:** Author affiliations: Centre Pasteur du Cameroun, Yaoundé, Cameroon (E. Marion, J. Landier, P. Boisier, S. Eyangoh);; Université d’Angers, Angers, France (E. Marion, L. Marsollier);; Institut Pasteur, Paris, France (J. Landier, A. Fontanet);; Institut de Recherche pour le Développement, Yaoundé (P. Le Gall);; Université de Nantes, Nantes, France (J. Aubry);; Hôpital de District de Bankim, Bankim, Cameroon (N. Djeunga);; Aide aux Lépreux Emmaüs Suisse, Yaoundé (A. Umboock)

**Keywords:** Buruli ulcer, Mycobacterium ulcerans, Cameroon, heteroptera, Bankim, Mycobacterium infections/epidemiology, tuberculosis and other mycobacteria, bacteria, letter

**To the Editor:** Buruli ulcer disease (BU) is a necrotizing skin disease caused by *Mycobacterium ulcerans* that affects mostly children in humid, tropical areas (*1*). The exact mode of *M. ulcerans* transmission remains unclear, although the role of water bugs has been supported by various observations and experimental studies (*2*,*3*). We report the identification of a new BU-endemic area in Cameroon, the Bankim district, and specify ecologic and clinical characteristics of *M. ulcerans* infection in this area. These characteristics hint at the possible role of environmental changes (building of a dam several years ago) in the expansion of BU in this area.

Since 1969, only 1 BU-endemic area in Cameroon has been described: the Nyong River basin, where equatorial forest predominates (*4*). In 2004, clinically suspected cases of BU in the district of Bankim have been reported (*5*). This region differs from the first BU-endemic area by geography and climate. Representing a transition between forested south and savanna north, this area has benefited from the building of a dam on the Mape River in 1989, which created an artificial lake of 3.2 billion m^3^ capacity.

From January 2007 through June 2009, all cases of skin lesions evocative of active BU were recorded as BU probable cases according to World Health Organization guidelines (*6*). During this period, 195 clinically suspected cases were reported from the Bankim health district (Figure). The overall median age for these 195 patients was 19.5 years (interquartile range 10–37 years). No significant difference in age was found according to gender, but a significant trend of decreasing overall median age was found (20 years in 2007 to 12 years in 2009. The most frequent type of lesion was ulcer. Since March 2009, the Centre Pasteur of Cameroon has performed laboratory confirmation for suspected BU cases: microscopic examination for acid-fast bacilli, culture, and *M. ulcerans* DNA detection by PCR (*6*). From April through June 2009, of 34 consecutive samples tested in the reference laboratory, 10 were positive for *M. ulcerans* by at least microscopy and PCR.

**Figure:**
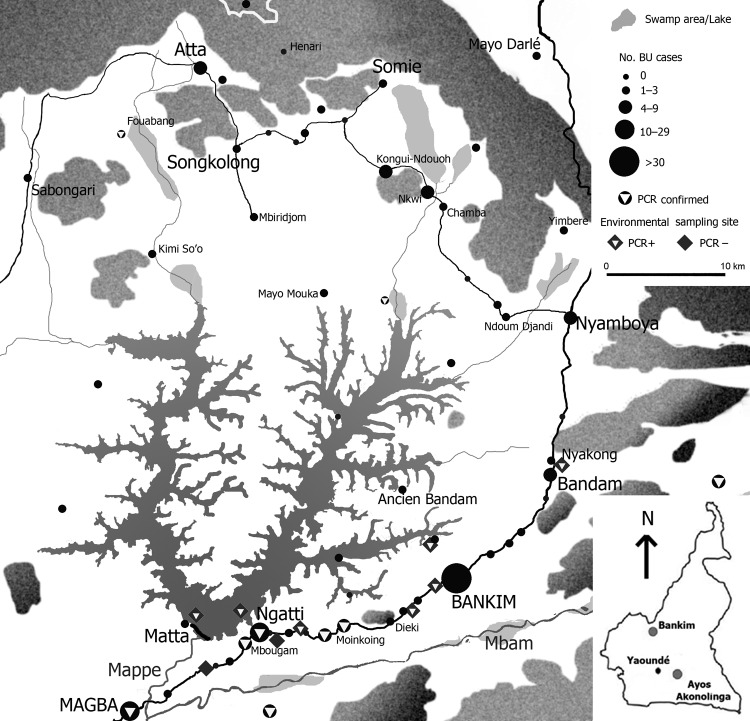
Distribution of Buruli ulcer (BU) patients reported January 2007–June 2009, and of water bodies with aquatic bugs harboring *Mycobacterium ulcerans,* Cameroon. Inset, Bankim area. A color version of this figure is available online (www.cdc.gov/EID/content/17/3/551-F.htm).

Whether BU is emerging in Bankim or is just a newly recognized preexisting disease is difficult to establish. However, that the incidence of BU in the region is increasing is unquestionable. The decreasing median age of patients since 2007 might be consistent with emergence of BU as a new disease in Bankim. This observation could suggest either an increasing level of acquired immunity in the population, leading to protection correlated with age, or the expansion of risky sites for human infection with *M. ulcerans*.

During 1 week in January 2008, water bugs were collected from the artificial lake and water bodies located within or close to each community. A previously described sampling method was used (*2*). To detect *M. ulcerans* DNA, we pooled the insects per family in groups of up to 10. Moreover, 99 members of the families Belostomatidae and Naucoridae were kept alive for saliva collection (*2*). The DNA of insect pool homogenized tissues and individual saliva samples were purified. We then searched for *M. ulcerans* molecular signatures (*2*). Among 1,349 insect specimens, 8 from the aquatic Heteroptera families were identified, and 12 (5%) of 244 insect pools were *M. ulcerans* positive. *M. ulcerans*–positive saliva was found in 11 (18%) of 61 insects in the family Belostomatidae and in 3 (8%) of 38 in the family Naucoridae. Water bodies where *M. ulcerans*–positive insects were collected are shown in the Figure.

The emergence of BU may be a consequence of the marked changes in the environment caused by the building of the dam. Elsewhere, human environmental modifications such as construction of dams have been linked with increased incidence of BU (*1*). The main visible environmental effect is the large amount of flooded farmland. According to the seasons, the reservoir margins change the milieu of swamps and meadows. All these modifications affect plant and animal resources in the reservoir area by favoring rapid growth of aquatic macrophyte populations during reservoir filling, thus providing breeding sites for insects and leading to the extinction of area-endemic species and creation of new niches (*7*). These changes might favor development of *M. ulcerans* in biofilms on aquatic plants, which are then ingested by herbivorous animals, which are further prey for water bug predators, hosts, and possible vectors of *M. ulcerans* (*8*,*9*). The water bugs that were most frequently trapped and colonized by *M. ulcerans* (families Belostomatidae, Naucoridae, Nepidae, Notonectidae) are carnivorous and able to bite humans (*10*).

Our study confirms expansion of BU in Cameroon. To facilitate detection of new BU foci, and to improve patient treatment (medical, surgical, rehabilitative), health care workers involved in tuberculosis/leprosy control programs should be educated about BU.
